# Feasibility of Total Endovascular Repair of the Aorta in Patients with Acute Type A Aortic Dissection: Morphological Analysis of 119 Patients

**DOI:** 10.3390/jcm12175615

**Published:** 2023-08-28

**Authors:** Wael Ahmad, Mark Liebezeit-Sievert, Moritz Wegner, Anastasiia Alokhina, Thorsten Wahlers, Bernhard Dorweiler, Maximilian Luehr

**Affiliations:** 1 Department of Vascular and Endovascular Surgery, Faculty of Medicine, University Hospital Cologne, University of Cologne, 50937 Cologne, Germanybernhard.dorweiler@uk-koeln.de (B.D.); 2 Department of Cardiothoracic Surgery, Heart Centre, Faculty of Medicine, University Hospital Cologne, University of Cologne, 50937 Cologne, Germanymaximilian.luehr@uk-koeln.de (M.L.)

**Keywords:** aortic dissection, stent graft, entry tear

## Abstract

(1) Background: This study aimed to morphologically analyze acute type A aortic dissection (aTAAD) patients for potential endovascular treatment candidates. The objective was to specify requirements for aTAAD endovascular devices. (2) Methods: A single-center retrospective analysis included aTAAD patients who underwent open surgical repair between November 2005 and December 2020. Preoperative CTA scans were used for morphological analysis, assessing endovascular repair eligibility. Statistical tests were performed. (3) Results: A total of 129 patients with aTAAD were studied, with 119 included. Entry tear (ET) locations were identified, mainly in the aortic root, 20 mm above the sinotubular junction (STJ) and within the ascending aorta (20 mm above STJ to −20 mm before the brachiocephalic trunk). Endovascular treatment was deemed feasible for 36 patients, with suggested solutions for the aortic arch and descending aorta. Significant differences were observed between eligible and noneligible groups for aortic diameter, false lumen diameter, distance between STJ and entry tear, and more. Dissection extension showed no significant difference. (4) Conclusions: Morphological analysis identified potential aTAAD candidates for endovascular treatment, highlighting differences between eligible and noneligible morphologies. This study offers insights for implementing endovascular approaches in aTAAD treatment and emphasizes the need for research and standardized protocols.

## 1. Introduction

Acute type A aortic dissection (aTAAD) poses a severe health threat, necessitating immediate surgical intervention to prevent major complications and potential fatality. Conservative management has been associated with mortality rates of up to 70% [1]. A variety of surgical techniques, each varying significantly in their extent, are utilized to address aTAAD, with perioperative mortality oscillating between 6% and 20% [2,3,4].

The advent and evolution of endovascular treatment strategies has provided a pathway for managing pathologies of the descending thoracic aorta. Thoracic endovascular aortic repair (TEVAR) has become a common practice, demonstrating improved outcomes when compared with open repair (OR) [5]. Similarly, the treatment of complicated type B aortic dissection (TBAD) is typically conducted using TEVAR or hybrid procedures that include aortic arch debranching, thus ensuring an adequate landing zone for thoracic stent grafts [6,7].

Given these encouraging results, endovascular treatment strategies for patients suffering pathologies involving the aortic arch, such as degenerative aneurysms or non-A-non-B aortic dissections, which are considered unfit for OR due to age, frailty, severe comorbidities, or prior cardiac or aortic surgery requiring sternotomy, have been developed [8,9,10,11,12]. Over the past decade, in order to reduce invasiveness, morbidity, and mortality in this specific subset of patients, endovascular approaches treating pathologies in the aortic arch have been investigated with promising results [13]. Evidence regarding these techniques in the aortic arch is usually limited to retrospective studies and prospectively collected data comparing outcomes with those of OR, and those using different endovascular solutions between each other are lacking. Long-term durability is unknown. Thus, standardized protocols and guideline recommendations have yet to be established.

To date, there has been a significant proportion of patients with aTAAD being turned down for OR due to high surgical risk, even in high-volume centers [14], and outcomes of OR in the elderly and morbid remain worse [15]. This led to descriptions of further expanding endovascular aortic repair in order to transfer the encouraging results witnessed in the aortic arch to the ascending aorta and aTAAD [16,17], and a first experience has been gained in carefully selected patients, limited to small case series in an experimental setting [18,19,20].

Although large entry tears and ascending aortic diameters, involvement of the aortic root or the coronary arteries, and the time required to manufacture patient-specific custom-made devices will hamper the applicability of endovascular treatment of aTAAD, valve-carrying conduits and stent grafts designed to meet the needs of the ascending aorta may complement the aortic specialists’ toolbox in well-selected patient cohorts in the future [21]. 

The objective of this study was to conduct an in-depth morphological analysis of patients with aTAAD, identifying potential candidates for endovascular treatment. We aimed to delineate how this treatment could be implemented and the specifications necessary for off-the-shelf devices designed for aTAAD treatment.

## 2. Materials and Methods

### 2.1. Study Cohort and Design

This single-center, retrospective analysis included patients diagnosed with aTAAD who underwent open surgical repair between November 2005 and December 2020. All patients underwent preoperative ECG-gated computed tomographic angiographic scans (CTA) with arterial-phase intravenous contrast injections and a maximum slice thickness of 3 mm. We excluded patients who had previously undergone ascending aorta or aortic arch repair and those for whom a CTA scan was unavailable.

Each patient’s diseased aortic segments underwent a detailed morphological analysis, and their eligibility for total endovascular repair procedure was assessed. When endovascular treatment was feasible, we delineated the possible endovascular approach. This study was conducted in compliance with the Declaration of Helsinki and was approved by our institutional ethics committee: Ethics Committee of the University of Cologne with code number 20-1212 on 26 August 2020.

### 2.2. Endovascular Treatment Eligibility Criteria

The CT angiographies’ morphology was analyzed to assess eligibility for endovascular treatment, considering the location and length of the entry tear (ET), potential proximal and distal landing zones, their diameters, the length and diameter of the supra-aortic arteries (if a total endovascular repair is required), presence of aneurysms, and the extent of the dissection in the aorta and its branches. The aim of the endovascular solution was to cover the entry tear(s) and to land in at least 20 mm of a not-diseased Aorta. If only 10 mm was available, we suggested a valve-carrying stent graft (TEVAR+TAVI: Trans-catheter Aortic Valve Implantation or TAVR (Transcatheter Aortic Valve Replacement)). Combining the concepts of TEVAR and TAVI, a valve-carrying stent graft would be designed to address pathology in the thoracic aorta that might also involve or be adjacent to the aortic valve, like type A aortic dissection extending to the aortic root.

This hybrid device would provide structural support (like TEVAR) to an affected segment of the aorta while also replacing a malfunctioning aortic valve or using the valve as a proximal sealing zone for the TEVAR.

An “endobental” procedure, defined as valve-carrying stent grafts with two fenestrations/ branches for the coronary arteries, was suggested if the landing zone was less than 10 mm and/or the false lumen had extended to the level of the coronary arteries or the aortic valve.

The classic Bentall procedure is an open-heart surgical procedure, which involves replacing the aortic valve, the ascending aorta, and the aortic root, while reimplanting the coronary arteries into the graft. It is often utilized for conditions such as aortic root aneurysms or aortic dissection involving the ascending aorta and the aortic root. 

When we talk about an “endovascular Bentall operation”, the following steps should be considered:

1. Endovascular Aortic Valve Replacement:
  Similar to TAVI/TAVR), a catheter would deliver a prosthetic valve to replace the patient’s aortic valve. This part of the procedure is already established and commonly performed for aortic stenosis.
2. Endovascular Aortic Root and Ascending Aorta Replacement:
  This would be the most challenging part. The graft would need to be designed in such a way that it could be anchored securely without risking occlusion of the coronary arteries. It would also need to be constructed in a way that the coronary arteries could be reconnected to the graft.
3. Coronary Ostia Reconnection:
  This remains a significant hurdle for an endovascular Bentall procedure. One potential solution would be some form of branched or fenestrated stent graft that has openings or branches aligned with the coronary ostia. Another option could be hybrid procedures where limited open surgery is used to connect the coronary arteries to the stent graft.

### 2.3. Data Collection

Measurements were taken in multiplanar reconstruction in the plane perpendicular to the manually corrected local aortic centerline using AGFA Impax EE (Version 20200429_0936, AGFA HealthCare, Mortsel, Belgium), including the following:

Distance between ET and sinotubular junction (STJ).
Length of ET.
Diameter of aorta and of true and false lumen.
Proximal and distal ascending aorta.
Aortic arch.
Possible distal landing zone.
Length and diameter of the supra-aortic arteries.
Diameter of the aortic annulus.

The suitability for an endovascular solution was assessed interdisciplinary by experienced vascular and cardiac surgeons.

### 2.4. Statistical Analysis

Data are expressed as median and interquartile range (IQ) for nonparametric data and as mean with standard deviation for parametric data. Mann–Whitney tests for independent samples were used to compare the continuous variable and chi-square tests to compare the categorical variable. A *p*-value < 0.05 was regarded as significant.

All statistical analyses were performed using SPSS 29.0 (IBM Corp., Armonk, NY, USA).

## 3. Results

### 3.1. Patient Characteristics

Over the study period, 129 patients were diagnosed with aTAAD. Ten patients were excluded due to subpar preoperative CT scan quality, preventing accurate aortic morphology measurement and assessment.

### 3.2. Entries’ Locations

**Figure 1:**
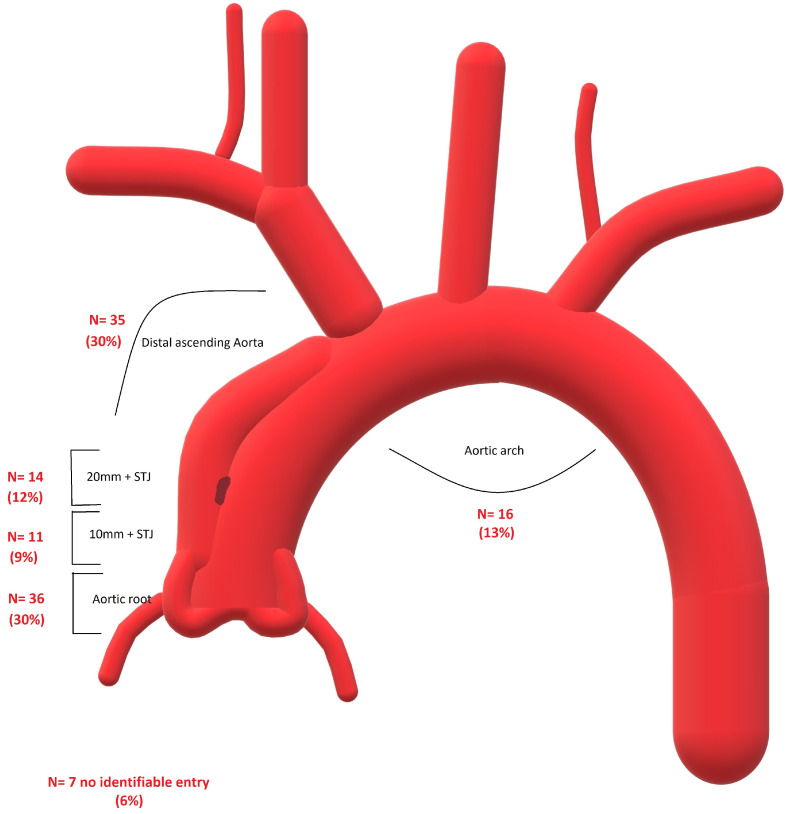
Overview of the location of the entry teats in the studied group.

Among the patients, 36 had the ET in the aortic root (from the aortic valve reaching the STJ). In the 20 mm above the STJ, 25 ETs were identified. We found 35 ETs in a coverable zone of the ascending aorta (20 mm above STJ until -20 mm before the brachiocephalic trunk (BCT)). The remaining ETs were situated in the aortic arch (6 at BCT, 4 at the left common carotid artery (LCCA), and 6 at the left subclavian artery (LSA)). In seven cases (two intramural hematomas), no ET was visible on the CT scan. Figure 1 provides an overview of the ETs.

### 3.3. Eligibility for Endovascular Treatment

**Figure 2:**
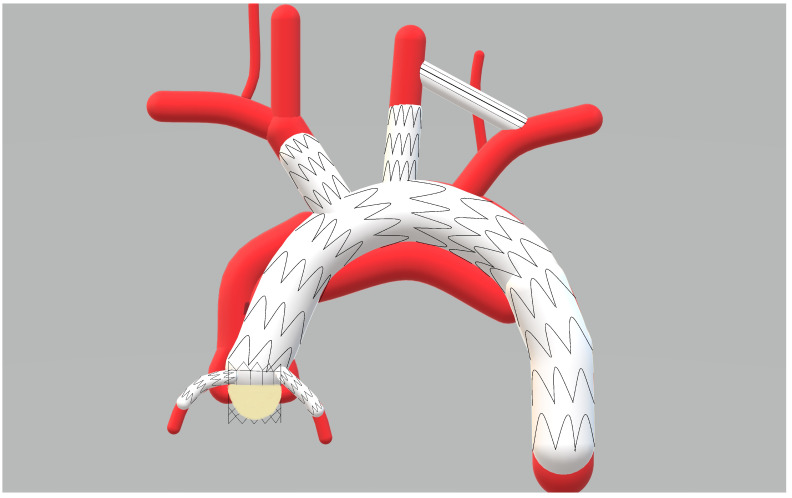
The endovascular management with a valve-carrying fenestrated stent graft (for coronary and supra-aortic arteries).

We identified total endovascular repair as a potential alternative to the open procedure in 36 patients. The suggested endovascular solutions are summarized in Table 1. The Society for Vascular Surgery and Society of Thoracic Surgeons’ reporting standards for type B aortic dissection were employed to report the outcomes. An endobental procedure was needed in 25 cases. This was required due to the extent of false lumen in the coronary arteries and/or to the aortic valve. Due to the extent of the dissection, additional endovascular interventions in the aortic arch and descending aorta, e.g., B/FTEVAR (branched/fenestrated TEVAR) or ChTEVAR (Chimney-TEVAR), were required. Figure 2 provides a suggested endovascular management with a valve-carrying fenestrated stent graft (for coronary and supra-aortic arteries).

Only three patients could be treated with a plain TEVAR in the ascending aorta. 

The mean aortic diameter at the proximal landing zone was 34 mm (SD ± 3 mm) and at the distal landing zone 33 mm (SD ± 3 mm).

### 3.4. Comparison between the Eligible and Noneligible Morphologies

Table 2 illustrates the measurements performed at the delineated locations, demonstrating the differences between the endovascular eligible morphologies and non-eligible ones. Among the examined parameters, several statistically significant differences were observed between the groups. Notably, the aortic diameter at STJ was significantly larger in the noneligible group (median 32 mm) than the eligible group (median 29 mm) (*p* = 0.001). Similarly, the diameter of the false lumen at the STJ and at 10 mm and 20 mm above the STJ was significantly greater in the noneligible group than in the eligible group (*p* = 0.001). Furthermore, the distance between the STJ and entry tear was significantly shorter in the noneligible group (median 1 mm) than the eligible group (median 36 mm) (*p* = 0.001). Additionally, the length of the entry tear was significantly greater in the noneligible group (median 18 mm) than the eligible group (median 12 mm) (*p* = 0.023).

The distribution of ET also differed significantly between the two groups. We observed a considerably higher prevalence of ET located at the aortic root in the not eligible than the eligible group (41% vs. 5%, *p* 0.001). Conversely, the eligible group demonstrated a greater occurrence of ET in the segment positioned 20 mm above STJ and 20 mm before BCT when compared with the noneligible group (47% vs. 22%, *p* < 0.005). These findings suggest distinct patterns of ET distribution in the two groups under consideration. 

No difference in the extension of dissection was noted (*p* 0.107). The most extensive extension reached A11 (until the external iliac artery) in 38% of the noneligible group and 30% of the eligible group (*p* 0.588).

The most frequent cause of the ineligibility was the aneurysmal dilatation of the ascending aorta (>50 mm) in 37 cases. The unfavorable position of ET at the coronary arteries (*n* = 15) or at the aortic valve (*n* = 10) was the second most frequent cause. In eight patients, the location of ET at the coronaries was accompanied by aneurysmal disease in the ascending aorta. Other sources of noneligibility were true lumen collapse in the ascending aorta (*n* = 7), retrograde A dissection after TEVAR, and extension of the dissection to the supra-aortic arteries (*n* = 1).

## 4. Discussion

Despite significant advancements in the management of aortic pathologies, aTAAD remains a crucial clinical challenge. The urgency of treatment is juxtaposed with the heightened risks that traditional surgical methods present, particularly in high-risk patient populations. The perioperative mortality of the classic open repair in those patients could reach 20% [2,3,4]. The overall prevalence of severe neurological events such as permanent stroke and spinal cord injury ranges between 7–18% in most single-center series in patients undergoing open surgery (frozen elephant trunk) for aTAAD [22,23,24]. 

As a comparison, the neurological complications, including stroke and spinal cord ischemia, have a reported incidence of 3–10% and 2.5–8%, respectively, after TEVAR in the descending aorta [25,26,27]. 

The inspiring success of endovascular techniques in treating the descending thoracic aorta ignites hope for its application in aTAAD. However, the complexity and heterogeneity of aTAAD cases necessitate an exhaustive understanding of the disease’s morphology. Factors such as the location and size of tears, extent of dissection, and the involvement of vital aortic branches profoundly influence treatment decisions. Additionally, as the ascending aorta holds a central position in the heart’s hemodynamics, managing dissections here requires meticulous strategies and advanced devices. This study takes a step into the challenging terrain of identifying suitable aTAAD patients for endovascular treatments, looking into the specificities required for generic devices and illuminating possible paths forward in this evolving therapeutic domain.

The idea of the total endovascular aortic repair in patients with type A aortic dissection is not new, and many studies and case presentations reported the implementation of the endovascular therapy in this considerable aortic disease [18,20,28]. 

The results of our study identify 36 patients (30%) with an acute type A aortic dissection eligible for total endovascular repair, with different suggested endovascular solutions based on specific morphological characteristics. In some cases, an “endobental” procedure, involving valve-carrying stent grafts with fenestrations/branches for the coronary arteries, was recommended for cases with limited landing zones. However, the applicability of endovascular treatment for aTAAD is limited by factors such as large entry tears, ascending aortic diameters, involvement of the aortic root or coronary arteries, and the time required to manufacture patient-specific custom-made devices.

Comparison between eligible and noneligible morphologies revealed significant differences in various parameters, including aortic diameter, false lumen diameter, distance between entry tear and sinotubular junction, and length of the entry tear. Notably, the aortic diameter at the sinotubular junction was significantly larger in the noneligible group, suggesting that patients with larger aortic diameters may not be suitable candidates for endovascular treatment.

The causes of noneligibility for endovascular treatment were mainly attributed to aneurysmal dilatation of the ascending aorta, unfavorable positioning of the entry tear at the coronary arteries or aortic valve, true lumen collapse in the ascending aorta, retrograde A dissection after TEVAR, and extension of the dissection to the supra-aortic arteries.

In their feasibility study, Kreibich et al. [17] screened patients with acute type A aortic dissection for anatomic feasibility of ascending aortic endovascular treatment with a valve-carrying conduit. Their main finding was that in 113 patients (68%), the entry was in a coverable zone in the ascending aorta with sufficient proximal and distal landing zone or in more distal aortic segments. However, detailed information about the location of ET in the aortic arch as well as the suitability for total endovascular arch repair is missing.

In their recently published work, Kern et al. [29] assessed the CTA-based anatomical suitability of currently manufactured stent grafts, as well as two embodiments of valve-carrying devices in 112 patients with type A aortic dissections. They found an anatomical feasibility ranging from 4% to 21%. For the valve-carrying conduits, anatomical feasibility was between 31% and 80%.

As we can see, the feasibility for the valve-carrying conduits was significantly higher in the above-mentioned studies than in ours. A possible rationale in our study could be the exclusion of patients with ET extending to the coronaries or the aortic valve (*n* = 25). Assuming the possibility of treating these cases with the valve-carrying conduits, the feasibility would reach 51%, close to those reported above.

Although this study provides valuable insights into identifying potential candidates for endovascular treatment of aTAAD, there are certain limitations that need to be acknowledged. First, the study is retrospective and single-center, which may limit the generalizability of the findings. Prospective multicenter studies with larger patient cohorts would provide more robust evidence for the effectiveness and applicability of endovascular treatment in aTAAD. Second, long-term follow-up data are lacking, and the durability of endovascular solutions remains uncertain. Further studies with extended follow-up periods are necessary to assess the long-term outcomes and complications associated with endovascular treatment. Moreover, developing a device or system to perform the entire Bentall procedure endovascularly would require significant technological advancements. It would need to accommodate the anatomical and functional challenges of replacing the aortic root and valve and ensuring the coronary arteries remain patent.

This should be supported with precise imaging modalities like image fusion, real-time imaging techniques such as advanced fluoroscopy and intravascular ultrasound (IVUS), and possibly even augmented reality systems.

Despite these limitations, this study contributes to the growing body of evidence supporting the potential benefits of endovascular treatment in selected patients with aTAAD. Identifying appropriate candidates for endovascular therapy based on morphological analysis is crucial for achieving optimal outcomes and reducing the invasiveness and mortality associated with aTAAD treatment. As endovascular techniques continue to evolve and advancements are made in device technology, the applicability of endovascular treatment for aTAAD may expand to include a broader range of patients in the future. Nevertheless, it is imperative to proceed with caution and prioritize patient safety when considering endovascular approaches for aTAAD, especially in cases with challenging anatomies and potential risks of device-related complications. Standardized protocols and guidelines should be established based on evidence from prospective studies to ensure the safe and effective implementation of endovascular treatment strategies for aTAAD.

## 5. Conclusions

The prospect of total endovascular aortic repair in patients with acute type A aortic dissection has been previously explored in various studies, underscoring its potential benefits. In our analysis of 119 patients, 30% were found to be eligible for endovascular repair based on specific morphological features. Some proposed endovascular strategies, such as the “endobental” procedure, highlight the evolving nature of these treatments. Nonetheless, challenges remain, including patient-specific factors like large entry tears, ascending aortic diameters, and the intricate nature of creating custom-made devices.

While our feasibility percentage for valve-carrying conduits was lower than some prior studies, this may be attributed to our exclusion criteria. If certain patients were considered, our feasibility could align more closely with previously reported rates.

Although technological advancements can provide new avenues, they also demand rigorous scrutiny for patient safety and device reliability. Our study offers valuable insights into potential endovascular treatments for aTAAD, emphasizing the significance of detailed morphological analyses for candidate identification. As endovascular technologies advance, the spectrum of treatable aTAAD patients may widen. Yet, patient safety remains paramount. Future endeavors should be steered by evidence from prospective studies, emphasizing the creation of standardized protocols to guarantee the optimal, safe application of these evolving endovascular strategies for aTAAD.

## Figures and Tables

**Table 1:** The suggested endovascular solutions of eligible morphologies.

Patient	Extension of Dissection *	Suggested Endovascular Procedure	Commentary	Entry Tear Location	DLZ
1.	A11	Endobental + B/FTEVAR + LSA Bp.	FL reaches aortic valve	Aortic Root	4
2.	A11	Endobental + B/FTEVAR + LSA Bp.	FL reaches coronaries	Aortic Root	4
3.	A11	Endobental+ ChTEVAR BCT/LCCA + LSA Bp.	FL reaches aortic valve	0–10 mm above STJ	4
4.	A11	Endobental + B/FTEVAR + LSA Bp.	FL reaches coronaries	0–10 mm above STJ	4
5.	A11	Endobental or: TEVAR + TAVI	FL reaches aortic valve	10–20 mm above STJ	5
6.	A11	Endobental+ ChTEVAR BCT/LCCA + LSA Bp.	FL reaches aortic valve	10–20 mm above STJ	4
7.	A8	Endobental + ChTEVAR + LSA-Bp.	FL reaches coronaries	10–20 mm above STJ	4
8.	A0	Endobental till BCT	FL reaches aortic valve	10–20 mm above STJ	0
9.	A11	Endobental + B/FTEVAR + LSA Bp.	FL reaches coronaries	BCT	4
10.	A10	Endobental + ChTEVAR + LSA-Bp.	FL reaches coronaries	BCT	4
11.	A9	Endobental + ChTEVAR + LSA-Bp.	FL reaches coronaries	BCT	3
12.	A11	B/FTEVAR, ChTEVAR + LSA Bp.		LCCA	4
13.	A9	Endobental + B/FTEVAR + LSA Bp.	FL reaches coronaries	LCCA	4
14.	B0	BTEVAR + LSA Bp.		LSA	5
15.	A11	B/FTEVAR or ChTEVAR (BCT + LCCA) + LSA Bp.		LSA	5
16.	A1	Endobental + B/FTEVAR + LSA Bp.	FL reaches coronaries	LSA	2
17.	A11	ChTEVAR + LSA-Bp.		LSA	4
18.	A2	Endobental + B/FTEVAR + LSA Bp.	FL reaches coronaries	LSA	5
19.	A10	Endobental + B/F TEVAR	FL reaches aortic valve	LSA	5
20.	A0	Endobental + TEVAR to BCT	FL reaches coronaries	20 above STJ −20 before BCT	0
21.	A9	Endobental + ChTEVAR +LSA-Bp.	FL reaches aortic valve	20 above STJ −20 before BCT	4
22.	A0	TEVAR 100 mm		20 above STJ −20 before BCT	0
23.	A2	TEVAR + BCT Periscope + RCCA-LCCA, LCCA-LSA Bps.		20 above STJ −20 before BCT	4
24.	A2	Endobental + B/FTEVAR + LSA Bp.	FL reaches coronaries	20 above STJ −20 before BCT	3
25.	A10	Endobental + B/FTEVAR + LSA Bp.	FL reaches coronaries	20 above STJ −20 before BCT	4
26.	A2	TEVAR 90 mm		20 above STJ −20 before BCT	0
27.	A11	BTEVAR + LSA Bp.		20 above STJ −20 before BCT	4
28.	A0	TEVAR 100 mm		20 above STJ −20 before BCT	0
29.	A11	B/FTEVAR, Debranching: LCCA-RCCA, Plug in BCT, TEVAR		20 above STJ −20 before BCT	1
30.	A10	Endobental + B/FTEVAR + LSA Bp.	FL reaches coronaries	20 above STJ −20 before BCT	5
31.	A3	ChTEVAR in BCT + RCCA-LCCA Bp. No ness. LSA Bp.		20 above STJ −20 before BCT	4
32.	A10	Endobental	FL reaches coronaries	20 above STJ −20 before BCT	
33.	A9	Endobental + ChTEVAR + LSA-Bp.	FL reaches aortic valve	20 above STJ −20 before BCT	4
34.	A0	Endobental till BCT	FL reaches aortic valve	20 above STJ −20 before BCT	0
35.	A10	Endobental + ChTEVAR (BCT/LCCA) + LSA Bp.	FL reaches aortic valve	20 above STJ −20 before BCT	4
36.	A9	Endobental + ChTEVAR + LSA-Bp.	FL reaches coronaries	20 above STJ −20 before BCT	4

B/FTEVAR: branched/fenestrated thoracic endovascular aortic repair, LSA Bp.: left common carotid artery–left subclavian artery bypass, FL: false lumen, STJ: sinotubular junction, TAVI: trans-catheter aortic valve implantation, BCT: brachiocephalic trunk, RCCA: right common carotid artery, LCCA: left common carotid artery, ChTEVAR: chimney-TEVAR, DLZ: distal landing zone. * According to the reporting standards of the Society for Vascular Surgery and Society of Thoracic Surgeons for type B aortic dissection.

**Table 2:** The differences in measurements between endovascular eligible morphologies and noneligible ones. Values in median and interquartile range.

	Eligible *n* = 36	Not Eligible *n* = 83	*p*
Diameter of the aortic annulus	15 (4)	15(4)	0.619
Distance highest coronary artery-STJ	8 (3)	7 (6)	0.398
Aortic diameter at STJ	29 (6)	32 (9)	0.001
Distance STJ–Entry	36 (37)	1 (22)	0.001
Distance STJ–BCT on the outer curve	98 (13)	102 (14)	0.003
Length of the entry tear	12 (12)	18 (16)	0.023
Diameter of TL at STJ	28 (9)	32 (20)	0.034
Diameter of TL at 10 above STJ	28 (11)	28 (30)	0.286
Diameter of TL at 20 above STJ	26 (14)	24 (34)	0.928
Diameter of FL at STJ	18 (11)	25 (18)	0.001
Diameter of FL at 10 above STJ	24 (11)	34 (18)	0.001
Diameter of FL at 20 above STJ	27 (8)	38 (15)	0.001
Length of the BCT	30 (5)	30 (5)	0.232
Diameter of the BCT	18 (4)	17 (4)	0.110
Diameter of the LCCA	10 (8)	10 (2)	0.232
Diameter of the LSA	13 (2)	13 (3)	0.372

STJ: sinotubular junction, BCT: brachiocephalic trunk, LCCA: left common carotid artery, LSA: left subclavian artery, TL: true lumen, FL: false lumen.

## Data Availability

The data presented in this study are available on request from the corresponding author. The data are not publicly available due to restrictions.
